# Alleviation of Morphine Withdrawal Signs but Not Tolerance by the Essential Oil of *Kelussia odoratissima* Mozaff.

**DOI:** 10.1155/2012/847096

**Published:** 2012-07-05

**Authors:** Mohammed Rabbani, Seyed Ebrahim Sajjadi, Azadeh Izadi

**Affiliations:** ^1^Department of Pharmacology and Pharmaceutical Sciences Research Center, Faculty of Pharmacy and Pharmaceutical Sciences, Isfahan University of Medical Sciences, Isfahan, Iran; ^2^Department of Pharmacognosy, Faculty of Pharmacy and Pharmaceutical Sciences, Isfahan University of Medical Sciences, Isfahan, Iran

## Abstract

The aim of the present study was to assess the effects of chronic and acute treatment of the essential oil (EO) of *Kelussia odoratissima* Mozaff. on the development of morphine tolerance and dependence in mice. Mice were rendered tolerant to and dependent on morphine by subcutaneous injection of morphine over a period of 5 days. Tolerance was assessed using the tail-pinch test and withdrawal signs of morphine were precipitated by injecting naloxone 2 h after the final morphine injection. Repeated injection of the EO of *K. odoratissima* (5 and 10 mg/kg) for 4 days significantly suppressed morphine-withdrawal jumps, a sign of the development of dependence to opiate as assessed by naloxone precipitation withdrawal on day 5 of testing. A single injection (25, 50, 100 mg/kg) of the EO on day 5, 1 h prior to morphine failed to produce any significant change in morphine withdrawal signs. Neither the acute nor the chronic administration of EO of the *K. odoratissima* did significantly influence the development of tolerance to the analgesic effect of morphine. Alleviation in morphine signs of withdrawal after chronic injection with *K. odoratissima* is indicative of reversal of neuronal adaptation that takes place during morphine presence in the brain.

## 1. Introduction

There are 275 genera and 2850 species in the Umbelliferae family [1]. *Kelussia odoratissima* Mozaff. is one of the latest species of the Umbelliferae family that is only found in Iran [2]. *K. odoratissima* is a monotypic and self-growing medicinal plant which is endemic of the restricted western parts of Iran and locally called “Karafs-e-koohi.” The aerial part of the plant is commonly used as a popular garnish and also as a folk medicine to treat hypertension and inflammation. Except the recent publications on the sedative [3] and antioxidant activity [4] of *K*. *odoratissima*, there are no other published pharmacological reports available.

Recent phytochemical study has revealed that the essential oil (EO) of the *K*. *odoratissima *consists of 27 components, representing 93.3% of the total oil. The main constituents of the EO are phthalides including 3-butylidene-4,5-dihydrophthalide (z-ligustilide) (85.9%), *cis*-3-butylidene phthalide (0.4%), and 3N butyl phthalide (0.3%). Other important identified components of the EO of *K. odoratissima* are *α*-copaene (1.4%) and *δ*-cadinene (0.7%) [3]. Ligustilide, the major component of the essence has been pharmacologically characterized for several actions [5]. Ligustilide has been shown to have antiasthmatic action, centrally acting muscle relaxant effect, effects on atria, and effects on central noradrenergic and/or GABA(A) systems [6–9]. In 2006, Cao et al. showed that Ligustilide caused vasodilation via inhibiting voltage-dependent calcium channels and receptor-mediated calcium influx and release [5].

Despite decades of research on opiates, our understanding of the mechanisms underlying physical dependence and withdrawal is still very limited. Among the neuronal systems that are studied after chronic morphine treatment, the voltage-dependent calcium channels have been the subjects of intensive research for last few years [10]. Considerable evidence has been published indicating that dihydropyridine-sensitive calcium channels play a role in physical dependence on morphine and other addictive drugs [10]. The number of dihydropyridine-sensitive binding sites in the CNS, thought to represent voltage-sensitive calcium channels, was demonstrated to increase in rats showing signs of morphine withdrawal [11]. Several authors have shown that organic calcium channel antagonists produce antinociception and enhance opioid-induced analgesia and hyperthermia [12]. Acute injection of L-type calcium channel blockers such as nimodipine, nifedipine, and nicardipine were found by us and others to protect against naloxone-precipitated morphine withdrawal in mice and rats [13–15].

Since phthalide make up the major component of the EO of the *K*. *odoratissima*, and these substances are known to bear calcium channel blocking property [5], this study was aimed to evaluate the effect of the EO of the plant on morphine tolerant and dependent mice. Support for the action of calcium channel antagonists in morphine dependent animals comes from numerous studies that mentioned before.

## 2. Material and Methods

### 2.1. Preparation of the Plant Material

The aerial parts of *K. odoratissima *were collected from the central Zagros region (Charmahal and Bakhtiari province) in western part of Iran in March 2010. The plant was identified at the Botany Department of Isfahan University, and a voucher specimen (no. 2022) was deposited at the Herbarium of the Faculty of Pharmacy and Pharmaceutical Sciences, Isfahan University of Medical Sciences, Isfahan, Iran.

### 2.2. Isolation of EO of *K. odoratissima*

The air-dried aerial parts of *K. odoratissima* were reduced to a course powder, and the oil was isolated by hydrodistillation using a Clevenger-type apparatus for 3 h according to the method recommended in the British Pharmacopeia [16]. The EO was stored in a sealed vial at 2–8°C before use. Appropriate amount of EO was diluted with normal saline (containing 2 drops of tween 80 per 10 mL) to give the suitable final concentration for pharmacological tests.

### 2.3. Animals

Male NMRI mice (Pasteur institute, Tehran, Iran) weighing 20–30 g were housed in cages of six at room temperature in a 12 h light-dark cycle. Food and water were available *ad libitum.* All experiments were conducted between 8:00 and 13:00 every day to avoid any temporal factor (e.g., circadian rhythm). Each animal was used for only one experiment condition. All experiments were carried out in accordance with the Guide for the Care and Use of Laboratory Animals at Isfahan University of Medicinal Sciences (2011).

### 2.4. Drugs

Morphine sulfate (Daru Pakhsh, Iran) 10 mg/mL ampoule was dissolved in saline to give a suitable final concentration, naloxone hydrochloride was purchased as an ampoule (0.4 mg/ml, Tolid Daru, Iran). Nifedipine was suspended in 4% DMSO in saline. Morphine was administered subcutaneously (s.c.) while naloxone and nifedipine were given intraperitoneally (i.p.). The control animals received the equivalent volume of vehicle in a volume of 10 mL/kg.

### 2.5. Morphine Withdrawal Syndrome

Group of 6 mice were chosen randomly for each dose of drugs. Morphine was injected s.c. twice daily at 8:00 and 16:00 for 5 days as described by Itoh et al. with the minor modifications [17]. Escalating doses, that is, first day (30 and 30 mg/kg at 8:00 and 16:00, respectively), second day (45 and 45), third day (60 and 60), fourth day (90 and 90) and fifth day (90 mg/kg at 8:00 only). The withdrawal signs were precipitated by injecting naloxone (5 mg/kg, i.p.) 2 h after the final injection of morphine. Immediately after a naloxone challenge, mice were individually placed in an observation box and withdrawal signs were evaluated during 30 min by counting the number of jumps and standings. Non-quantitative signs (diarrhea, hair raising, eye ptosis and tremor) were rated according to Gellert and Holtzman rating scale [18]. This scale consists of graded signs and checked signs. Graded signs were assigned a weighting factor from 1 to 4 which based on the frequency of their appearance.

### 2.6. Acute and Chronic Drug Administration

For acute treatment, the EO of *K. odoratissima* and nifedipine were administered 1 h after the final morphine injection. For chronic treatment, the EO of the *K. odoratissima* and nifedipine were injected 30 min prior to morphine morning injection for 4 days.

### 2.7. Tolerance Paradigms

Group of 6 mice were chosen randomly for each dose of drugs. The treatment schedule consisted of twice daily s.c injections of 20 mg/kg morphine at 8:00 and 16:00 for 4 days and 20 mg/kg only at 08:00 on the 5th day. For acute administration, the EO of *K. odoratissima*, nifedipine and vehicle were given 30 min before the last injection of morphine on day five. For chronic treatment, the EO of *K. odoratissima*, nifedipine, and vehicle were administered i.p 30 min before morning injections of morphine for 4 days. Control mice received the same volume of the vehicle.

Tolerance was assessed 30 min after the final morphine injection based on loss of the antinociceptive effect of morphine during 5 days, using the modified Haffner's method, the tail-pinch test [19]. In the tail-pinch assay, a flattened clip (about 10 mm wide) was placed approximately 1 inch from the base of a mouse's tail, and the response time of animal to the exerted pain was measured. The mice that responded to the clip placement by turning back and biting the clip within 15 s were used in this test. Drug-naïve mice responded to this pressure by immediately vocalizing and biting the clip. The mice treated by a single injection of morphine did not respond to the pain during 15 sec. The clip was never applied to the mouse tail longer than this. An animal that did not respond before 15 s (cut-off time) was assigned a latency of 15 s. Response time to pain was reduced after development of morphine tolerance on the 5th day.

### 2.8. Statistical Analyses

Quantitive data were assessed using *t*-test and one-way analysis of variance (ANOVA) with posthoc Newman-Keuls test and expressed as mean ± S.E.M. Nonparametric data scores were analyzed using Kruskal-Wallis ANOVA with Dunn's test and expressed as median ± interquartile ranges. In all comparisons, *P* < 0.05 was considered significant.

## 3. Results

### 3.1. Acute Effect of **K. odoratissima **on Morphine Dependence

**Figure 1:**
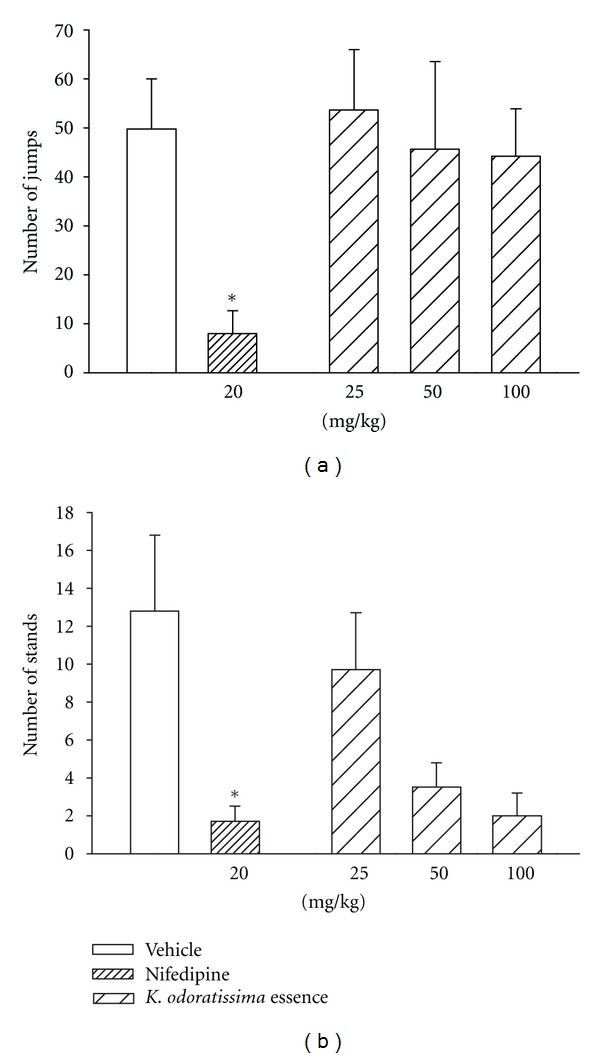
Effects of acute administration of different doses of the essential oil of *K. odoratissima *on the number of (a) jumps and (b) stands. Morphine was given in increasing doses over a period of 5 days. The different doses of *K*. *odoratissima*, nifedipine, and vehicle were injected in a single dose, 1 h after the final dose of morphine and 1 h prior to naloxone administration. The withdrawal signs were induced by naloxone (5 mg/kg) and recorded for 30 minutes. Results are the mean (±S.E.M.) from group of 6 mice. *P < 0.05 for comparison with vehicle-treated group.

In mice treated with morphine for 5 days, naloxone injections precipitated the standard behavioral signs of withdrawal. In drug naïve group, however, the injection of naloxone did not trigger behavioral changes (data are not shown). Acute administration of *K. odoratissima* at the doses of 25, 50, and 100 mg/kg did not exert significant effect on the number of jumps and stands (Figures 1(a) and 1(b)). Nifedipine (20 mg/kg) as a reference drug did, however, significantly reduce the number of jumps and stands (Figures 1(a) and 1(b)). 

Several nonquantitative withdrawal signs were assessed next. Acute injection of the plant essence at various doses was ineffective in blocking the above mentioned signs (Table 1). Nifedipine at 20 mg/kg, however, was only effective in significantly reducing the hair standing and tremor signs.

### 3.2. Chronic Effect **K. odoratissima ** on Morphine Dependence

**Figure 2:**
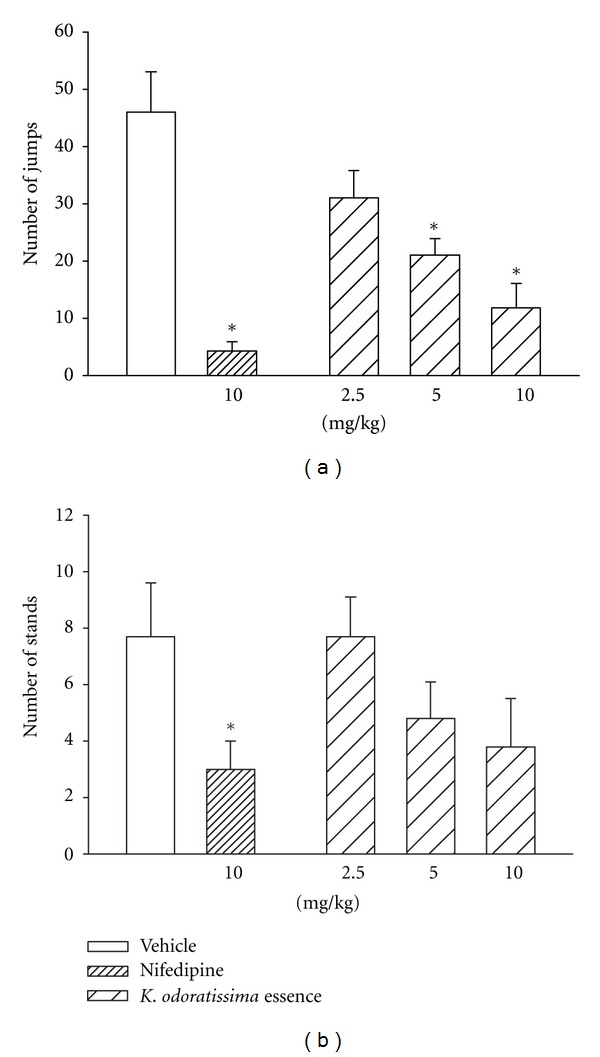
Effects of chronic administration of different doses of the essential oil of *K. odoratissima* on the number of (a) jumps and (b) stands. Morphine was given twice daily over a period of 5 days. The different doses of *K*. *odoratissima, *nifedipine, and vehicle were injected once a day during morphine injection at the morning for 4 days. The withdrawal signs was induced by naloxone (5 mg/kg) and recorded for 30 minutes. Naloxone was injected 2 hours after the final morphine injection. Results are the mean (±S.E.M.) from group of 6 mice. *P < 0.05 for comparison with vehicle treated controls.

Chronic injection of *K. odoratissima* for 4 days at doses of 2.5, 5, and 10 mg/kg was tested next. The plant essence at 5 and 10 mg/kg significantly reduced the number of jumps without affecting the number of stands. Chronic treatment with nifedipine at 10 mg/kg also significantly lowered the number of jumps (Figure 2(a)) without significantly changing the number stands (Figure 2(b)). 

Chronic treatment with the essence at various doses did not significantly change the qualitative signs of withdrawal (Table 2). Chronic nifedipine at 10 mg/kg, however, was only effective in blocking the tremor signs (Table 2).

### 3.3. Acute and Chronic Effects of **K. odoratissima **on Morphine Tolerance

**Figure 3:**
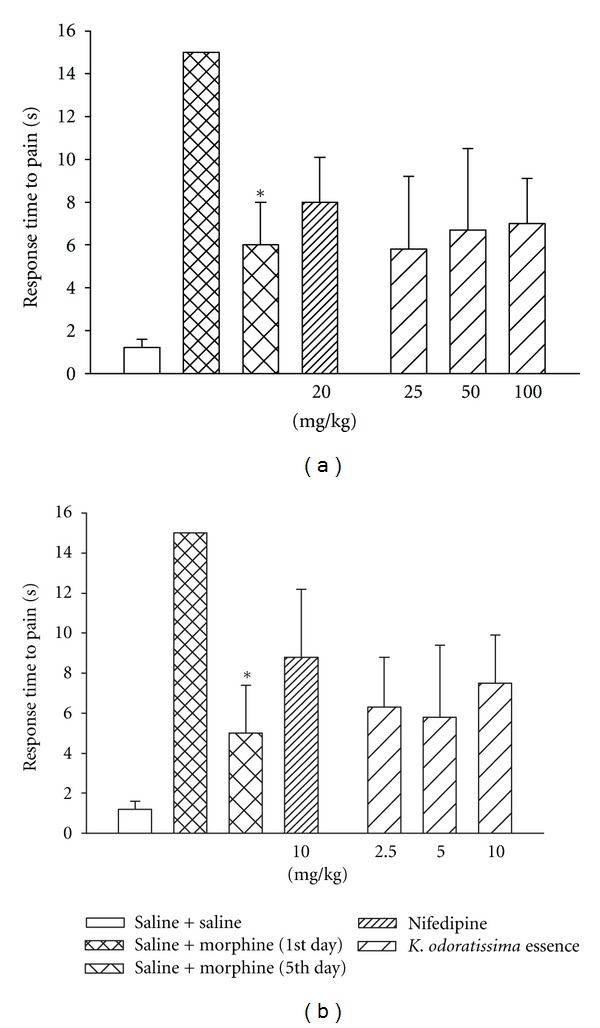
Effect of (a) acute and (b) chronic administration of different doses of the essential oil of *K. odoratissima *on the development of morphine tolerance in mice. Mice treated by a single injection of morphine (first day) did not respond to the pain during 15 sec (cut-off time). Morphine was given twice daily over a period of 5 days as described in the method section. In the acute administration different doses of *K*. *odoratissima, *nifedipine and vehicle were injected 30 min prior to the last injection of morphine in the morning of the 5th day. In the chronic administration different doses of *K*. *odoratissima, *nifedipine and vehicle were injected once a day during morphine injection in the morning for 4 days. Results are the mean (±SEM) from groups of 6 mice. *P < 0.05 for comparison with saline + morphine 1st day.

The tail pinch test was used to investigate the effects of acute and chronic injection of *K. odoratissima *on the development of morphine tolerance. In naive mice the response time to pain was 1.2 seconds (Figures 3(a) and 3(b)). As depicted in Figures 3(a) and 3(b), injection of a single dose of morphine at 20 mg/kg produced an antinociceptive effect that was observed by the lack of response to exerted pain during the cut-off time (15 s). The mean response time to pain was gradually shortened from 15 s on first day to 6 and 5 seconds on the 5th day in animal receiving saline and morphine which shows the development of tolerance (Figures 3(a) and 3(b)). Neither the acute nor the chronic treatment with plant essence had significant effect on the development of tolerance to morphine. Nifedipine also could not inhibit the development of morphine tolerance (Figures 3(a) and 3(b)).

## 4. Discussion 

The aim of this study was to evaluate the effect of the EO of *K. odoratissima* in morphine tolerant and dependent mice. In this study, morphine in increasing doses over a five-day period followed by naloxone produced a full-blown withdrawal syndrome. This program was used to evaluate the ability of the plant essence to inhibit withdrawal signs such as jumping, standing, diarrhea, hair raising, tremor, and eye ptosis. In the naloxone-precipitated withdrawal study, acute injection of *K. odoratissima*, 1 hour after the last morphine injection (1 hour before naloxone) did not significantly reduce any signs of withdrawal. Chronic treatment of *K. odoratissima* (5 and 10 mg/kg)however, significantly reduced the number of jumps, without affecting the other signs of withdrawal. Jumping is considered as an important sign for evaluating opiate dependence, though an effective drug should equally work against other signs of withdrawal.

The exact mechanism of action of the plant essence could not be predicted from these experiments. However, the fact that chronic treatment with the essence had reduced the number of jumps indicates some reversal of neuronal adaptation that had taken place during chronic morphine treatment. The EO of the *K*. *odoratissima *consists of 27 components, representing 93.3% of the total EO. The main constituents of the EO are ligustilide [3]. Ligustilide has been shown to block the voltage dependent calcium channels in rat mesenteric artery causing vasodilatation [5]. Chronic injection of morphine and other opioid agonists have been shown by several groups to produce an increase in brain calcium [10] and increase in the number of voltage-dependent calcium channels [11]. Abrupt cessation of morphine causes a massive influx of calcium that leads to withdrawal hyperexcitability [10]. Calcium channel antagonists during chronic morphine treatment have been shown to prevent naloxone-induced upregulation of calcium channel antagonists binding sites [12]. Furthermore, treatment with L-type calcium channel blockers such as nimodipine, nifedipine, and nicardipine were found by us and others to protect against naloxone-precipitated morphine withdrawal in mice and rats [13–15]. A possible explanation for the action of *K. odoratissima* EO could be drawn from its calcium channel blocking properties that helps preventing the neuronal calcium channel upregulation that occurs during repeated injection of morphine. 

Neither the acute nor the chronic injection of essence had significant effect on the development of tolerance to morphine. Unlike the lack of effect in development of tolerance, chronic treatment of the essence was effective in alleviating some signs of morphine withdrawal. Our data suggest the existence two possible mechanisms for the development morphine tolerance and dependence. There are number of studies in which the tested drugs have prevented the withdrawal hyperexcitability signs without affecting the development of tolerance. For example, diltiazem was shown to be effective in blocking morphine withdrawal signs [20] but failed to have significant effect on development of tolerance [21]. Similar results have been reported with other calcium channel antagonist such as nimodipine and nifedipine [22–25]. On the other hand, there are some studies that argue for an involvement of a common mechanism for tolerance and dependence to morphine. To reach an appropriate conclusion, one need to take to account the exact methodology (type of animals, dose regiments and the evaluation methods for tolerance and dependence) that has been used to make the animals tolerant and dependent. 

Finding a suitable drug to cure addiction has been one of the far reaching goals of scientists. Lack of success in finding synthetic chemical has inspired the investigators to search among the natural products. Variety of plant essence and extracts have been tested so far, some with effects and many without significant effects. For example, *Nepeta glomerulosa, Otostegia persica, Salvia leriifolia, Ferula gummosa, Panax ginseng, Withania somnifera, and Delphinium denudatum *[26–32]. In this study we showed that *K. odoratissima* could prevent one of the major signs of morphine withdrawal, that is, jumping. Although the essence of this plant does not change the pattern of tolerance development, it could be used in combination with other medications to alleviate morphine withdrawal hyperexcitability.

## Figures and Tables

**Table 1:** The effect of acute administration of the essential oil of the *K. odoratissima *on naloxone-precipitated withdrawal signs in morphine dependent mice.

Treatment	Urine volume (mL)	Eye ptosis	Hair standing	Tremor
Control	2 (1–3)	3.5 (2–4)	3 (2-3)	3 (2-3)
Nifedipine (20 mg/kg)	1 (1-1)	2 (2-2)	1.5 (1-2)^∗^	1.5 (1-2)^∗^
Essence (25 mg/kg)	1.5 (1–3)	2.5 (2-3)	2.5 (2-3)	2 (2-3)
Essence (50 mg/kg)	1.5 (1-2)	2 (2-3)	2 (1–3)	2.5 (2–4)
Essence (100 mg/kg)	2 (1-2)	3.5 (2–4)	3 (2-3)	3 (2–4)

Morphine was given in increasing doses over a period of 5 days as described in method section. The different doses of *K*. *odoratissima, *nifedipine, and vehicle were injected 1 h after the last dose of morphine (1 h before naloxone injection). The withdrawal signs were precipitated by naloxone. Withdrawal signs were observed for 30 min. ^∗^ *P* < 0.05 versus saline control using Kruskal-Wallis ANOVA followed by Dunn's test. The results are the median scores for withdrawal signs (± interquartile ranges in parenthesis).

**Table 2:** The effect of chronic administration of the essential oil of the *K. odoratissima *on naloxone-precipitated withdrawal signs in morphine-dependent mice.

Treatment	Urine volume (mL)	Eye ptosis	Hair standing	Tremor
Control	2 (2-2)	3 (1.5–3.5)	1.5 (1-2)	2 (2-3)
Nifedipine (10 mg/kg)	2 (1-2)	1.5 (1-2)	1 (1-2)	1 (1-1)^∗^
Essence (2.5 mg/kg)	2 (1-2)	2.5 (2-3)	2 (2-2)	2 (2-2)
Essence (5 mg/kg)	1.5 (1-2)	3 (2-3)	2 (1–3)	2 (1–3)
Essence (10 mg/kg)	1.5 (1-2)	2 (1-2)	2 (1-2)	1 (1-2)

Morphine was given in increasing doses over a period of 5 days as described in method section. Morphine was given twice daily over a period of 5 days. The different doses of *K*. *odoratissima, *nifedipine, and vehicle were injected once a day during morphine injection at the morning for 4 days. The withdrawal signs were precipitated by naloxone. Withdrawal signs were observed for 30 min. ^∗^ *P* < 0.05 versus saline control using Kruskal-Wallis ANOVA followed by Dunn's test. The results are the median scores for withdrawal signs (± interquartile ranges in parenthesis).
